# Explaining the trends and variability in the United States tornado records using climate teleconnections and shifts in observational practices

**DOI:** 10.1038/s41598-021-81143-5

**Published:** 2021-01-18

**Authors:** Niloufar Nouri, Naresh Devineni, Valerie Were, Reza Khanbilvardi

**Affiliations:** 1 Department of Civil Engineering, The City University of New York (City College), New York, NY 10031 USA; 2 NOAA/Center for Earth System Sciences and Remote Sensing Technologies (CESSRST), The City University of New York (City College), New York, NY 10031 USA

**Keywords:** Climate sciences, Natural hazards

## Abstract

The annual frequency of tornadoes during 1950–2018 across the major tornado-impacted states were examined and modeled using anthropogenic and large-scale climate covariates in a hierarchical Bayesian inference framework. Anthropogenic factors include increases in population density and better detection systems since the mid-1990s. Large-scale climate variables include El Niño Southern Oscillation (ENSO), Southern Oscillation Index (SOI), North Atlantic Oscillation (NAO), Pacific Decadal Oscillation (PDO), Arctic Oscillation (AO), and Atlantic Multi-decadal Oscillation (AMO). The model provides a robust way of estimating the response coefficients by considering pooling of information across groups of states that belong to *Tornado Alley*, *Dixie Alley*, and *Other States*, thereby reducing their uncertainty. The influence of the anthropogenic factors and the large-scale climate variables are modeled in a nested framework to unravel secular trend from cyclical variability. Population density explains the long-term trend in *Dixie Alley*. The step-increase induced due to the installation of the Doppler Radar systems explains the long-term trend in *Tornado Alley*. NAO and the interplay between NAO and ENSO explained the interannual to multi-decadal variability in *Tornado Alley*. PDO and AMO are also contributing to this multi-time scale variability. SOI and AO explain the cyclical variability in *Dixie Alley*. This improved understanding of the variability and trends in tornadoes should be of immense value to public planners, businesses, and insurance-based risk management agencies.

## Introduction

Tornadoes are one of the most devastating, severe weather events in the United States (U.S.) that always pose risks to human life and cause extensive property damage. Tornado outbreaks, defined as sequences of multiple tornadoes closely spaced in time, lead to the most number of fatalities and rank routinely among severe weather events that cause billion-dollar losses^1^. An average annual loss of $982 million is reported for U.S. tornado events based on insurance catastrophe data from 1949–2006, which clearly indicates their destructive nature^2^.

 Modern logging of tornado reports in the U.S. has begun in the 1950s, and the reports grew even more in the mid-1990s, mainly due to the increase in the detection of EF0–EF1 category tornadoes (weak tornadoes) after the installation of the NEXRAD Doppler radar system^3–10^. Other factors, such as better documentation, more media coverage, rise in the population, and storm chasing also contributed to this increased detection rate. Recent studies show that this secular trend (i.e., the long-term trend that is not due to seasonality) in the annual frequency of tornadoes has also been varying spatially. See, for example, Gensini and Brooks^11^ who showed that the temporal trends since 1979 have a significant spatial variability, and Moore^12^, who found that the number of tornadoes per year increased in the southeast U.S. and has decreased in the Great Plains and the mid-west regions. Studies by Farney and Dixon^13^ and Agee et al.^6^ show similar results.

**Figure 1:**
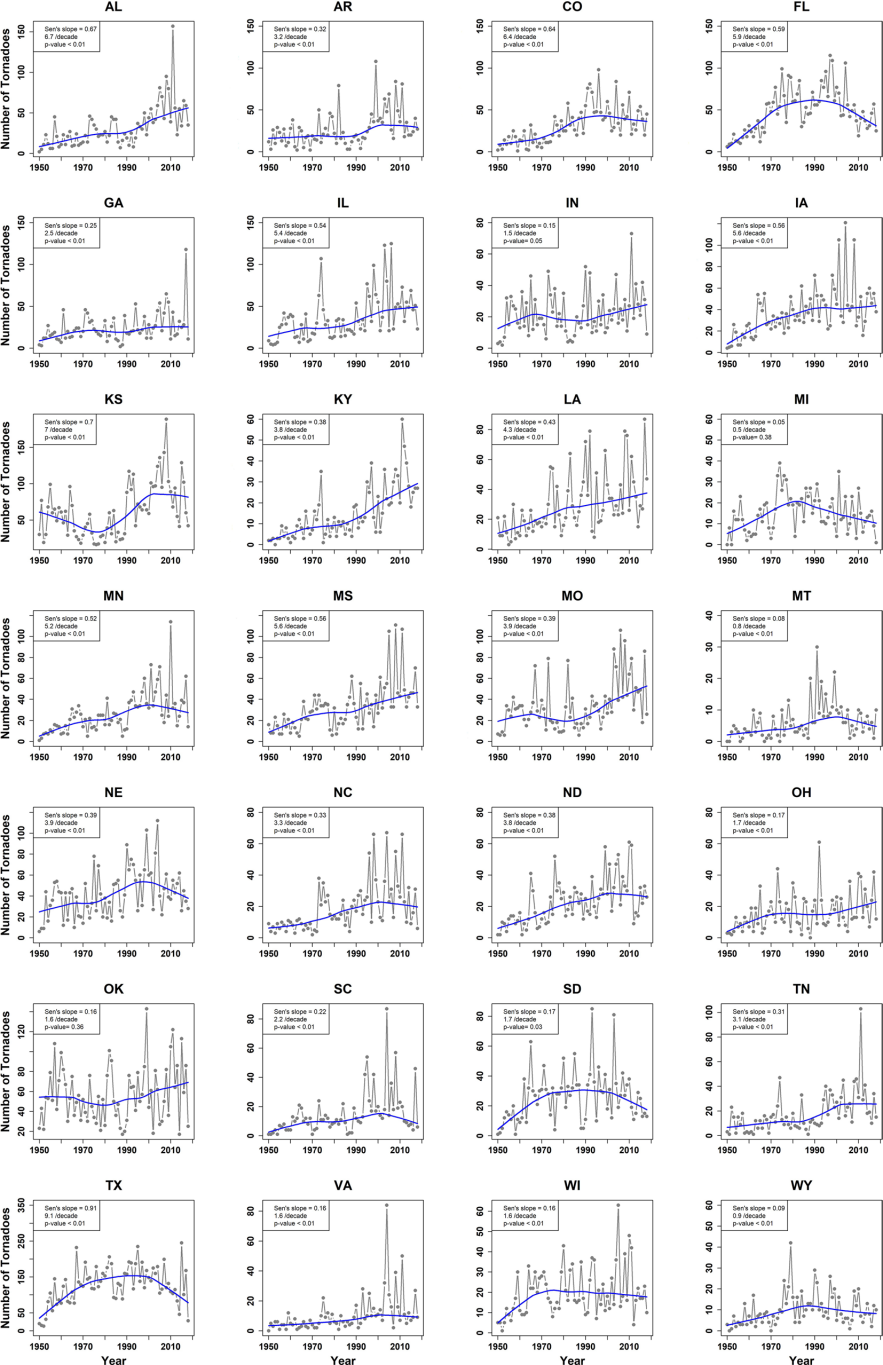
Annual number of tornado incidence in 28 major tornado-impacted states in the U.S. during 1950–2018. The blue line is locally-weighted polynomial fit^20^ using a smoothing span of 0.5. For each state, Mann–Kendall trend test is performed and the *p-value* of the test along with Sen's slope or the rate of change is presented.

Figure 1 presents the confirmation of trends in the annual frequency of tornadoes in 28 major tornado-impacted states in the U.S. Statistically significant secular trends (as estimated using the Mann Kendall Sen Slope^14^) are found in 26 states. Evidently, there also seems to be a step-change in many of these states since the 1990s, along with signs of cyclical trends in others (for example, Texas, South Dakota, and Florida). The inter-annual variability is also prominent and seems to have increased in recent decades. That the inter-annual variability in tornado counts relates measurably to El-Nino Southern Oscillation (ENSO), a large-scale climate teleconnection feature is not unknown. See, for instance, Cook and Schaefer^15^, Lee et al.^16^, Marzban et al.^17^, and Allen et al.^18^, among others. A recent investigation of the tornado activity in the southeast U.S. region noted an association with the North Atlantic Oscillation (NAO) in addition to ENSO and regional sea surface temperature anomalous patterns^19^.

**Figure 2:**
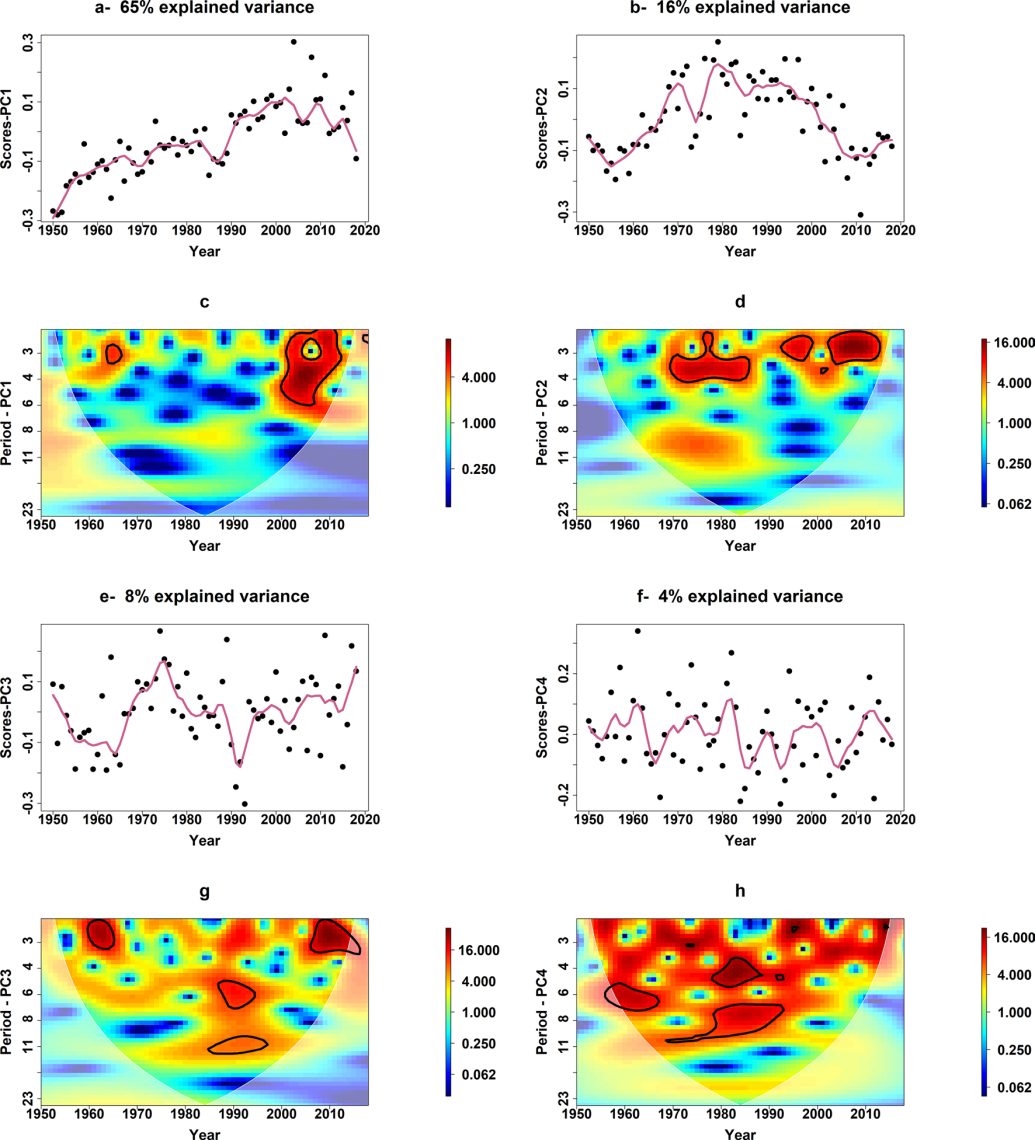
(**a**,**b**,**e**,**f**) represents the time series plots for the scores of the first four principal components. The pink line is the locally weighted smoothing line with span of 0.1. (**c**,**d**,**g**,**h**) is the wavelet power spectrum of the corresponding score. Higher wavelet power during a specific time shows notable frequencies in tornado activity. The areas with solid black line represent significant wavelet power at the 90% confidence interval. (Wavelet plots are created in R version 3.5.2 using biwavelet package^26^, https://CRAN.R-project.org/package=biwavelet).

A more in-depth examination that we conducted using robust principal components analysis and wavelet transformation to decipher the hidden structures in the time series of the tornados' annual frequency across the 28 major tornado-impacted states revealed systematic low-frequency oscillations beyond the inter-annual time scale. We show this in Fig. 2. The high-dimensional annual tornado frequency data (69 years of counts data for the 28 major tornado-impacted states) was first reduced to its dominant modes using robust principal components analysis^21^ (rPCA). The first four dominant modes (principal components or scores) explained up to 93% of the original data variance with the first mode capturing the secular trend in the data as a result of increased tornado detection. The other modes exhibited cyclical variability. Wavelet transformation^22–25^ of these principal components unveiled oscillatory features at the interannual to multidecadal time scales with much lower frequency signals in the third and the fourth modes. For example, the wavelet analysis of the first three principal components showed significant wavelet power (at the 90 percent confidence interval) occurring in period band of 2–5 years, which reveals notable variability in the data with a frequency of 2–5 years. This typically corresponds to low-frequency climate variability. The significant period band of 2–5 years occurred in mid 1960s and during 2000–2010 in the first PC, while in the second PC, the periodic activity was significant during 1970 to mid-80s, mid 1990s and 2000–2012. The third principal component also reveals similar period band which was notable in early 1960s and 2005–2015. The power spectrum plot of the third PC (2-g) showed a significant wavelet power at a period band of 5–10 years during mid-1980s to mid-1990s, which suggests decadal oscillatory behavior.

These preliminary inquiries and experiments prompted us to devise a comprehensive modeling approach to infer the key explanatory factors of the annual frequency of tornadoes across the major tornado-impacted states in the U.S. There is evidence of secular trend and cyclical variability, and this might vary spatially. Hence, there is a need to understand the effects of possible anthropogenic factors and large-scale climate teleconnections separately.

Anthropogenic factors such as the rise in population and the installation of better detection systems may have led to a monotonic increase or a step-change in the frequency of tornadoes. Hence, to explain the secular trend, we choose two indicators—the annual population density data (PD) for each state and a binary Doppler Radar Indicator (DRI) with zero from 1950–1990 and switches to 1 from 1991–2018—to factor in the installation of the NEXRAD Doppler radar system in the early 1990s. Large-scale climate oscillations may control the interannual to multidecadal variability seen in the data. Hence, to explain the cyclical trend, we factored in a suite of annual climate indices—El Niño/Southern Oscillation (ENSO)—through the Nino3.4 index, Southern Oscillation Index (SOI), North Atlantic Oscillation (NAO), Pacific Decadal Oscillation (PDO), Arctic Oscillation (AO) and Atlantic Multi-decadal Oscillation (AMO). We conducted a wavelet coherence analysis^27–29^ between the first four principal components described in Fig. 2 and these climate indices for additional validation of their selection. Evident joint variability is seen in the coherence plots (see Figure S2 in the supplemental material), indicating that the low-frequency oscillation of climate could drive part of the variability in the tornadoes' annual frequency. As mentioned before, these anthropogenic and climate effects can vary in space. Still, there is a possibility of some commonality in the impacts over specific regions, such as the *Tornado Alley*, *Dixie Alley*, etc.^3,30,31^.

These conditions inspired us to build nested models using a hierarchical Bayesian inference framework, which not only allows for a full uncertainty quantification but also its reduction by pooling information across appropriate classification of states. The hierarchical framework provides an elegant means of propagating the parameter uncertainty through appropriate conditional distributions. Further, noting that multiple climate and anthropogenic covariates influence tornadoes across a region (*Tornado Alley*, *Dixie Alley*, etc.) similarly, the hierarchical model provides pooling of this common information. This type of pooling reduces the equivalent number of independent parameters, resulting in lower uncertainty in parameter estimates. The models are explained in detail in the Methods section. Using this holistic modeling framework, for the first time, we explained the factors governing secular trends and cyclical variability in the annual frequency of tornadoes across the major tornado-impacted states in the U.S. The relative contribution of the anthropogenic and climate factors and their primary influence regions are discussed.

## Results

### Explaining the variance

**Figure 3:**
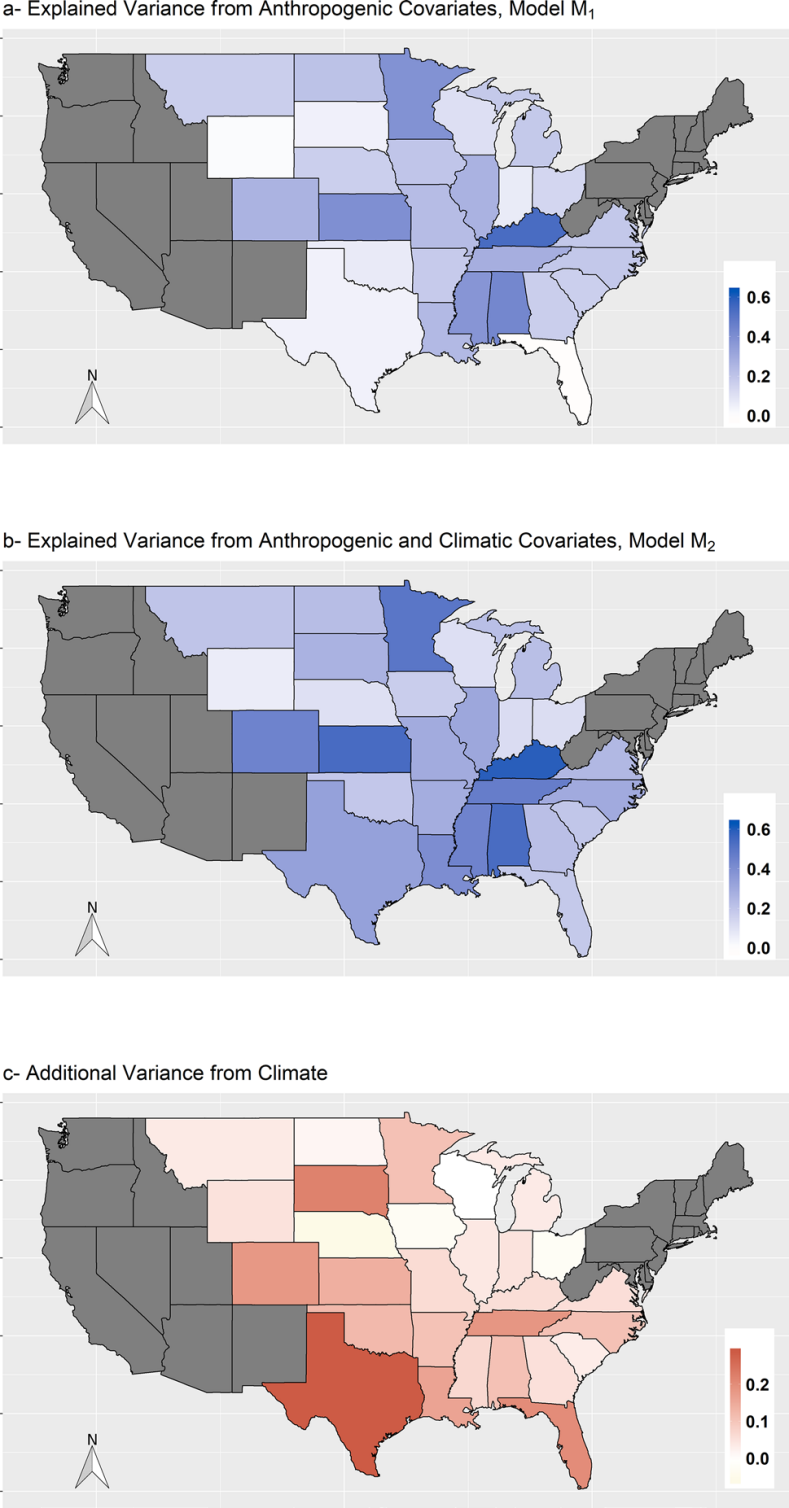
Spatial distribution of the explained variance from the nested models (M_1_ and M_2_). The explained variance, i.e., *R*^*2*^, is calculated based on sum of squared residuals, ${R}_{i}^{2}=1-\frac{\sum {({y}_{it}-{\widehat{y}}_{it})}^{2}}{\sum {({y}_{it}-{\stackrel{-}{y}}_{i})}^{2}}$ , where ${y}_{it}$ and ${\widehat{y}}_{it}$ are the observed and the predicted posterior median of the tornado counts in year *t* and state *i*, and ${\stackrel{-}{y}}_{i}$ is the mean of the observational data during the entire period. (Maps are created in R version 3.5.2 using the ggplot2 package^32^, https://CRAN.R-project.org/package=ggplot2).

Figure 3 presents the spatial distribution of the explained variance from the nested models (M_1_ and M_2_—see the Methods section for a description of these models). We first show the state-level *R*^2^ of M_1_, the model that uses PD and DRI as the anthropogenic covariates to understand their influence in producing the secular trend. The state-level *R*^2^ of model M_2_ that uses these two anthropogenic covariates as well as the climate covariates (SOI, NAO, the interaction between Nino3.4 and NAO, PDO, AMO, and AO) is shown next. The difference in the *R*^2^ between the two models is also presented. This difference map measures the additional explanation that the climate covariates provide in each state. Table S2 in the Supplemental Material provides the numerical details for each state.

The two anthropogenic covariates explained 17%, 28%, and 19% of the variance in the annual tornado frequency on average across *Tornado Alley*, *Dixie Alley*, and *Other States*, respectively. Greater than 30% variance in Kansas, Alabama, Mississippi, Kentucky, and Minnesota was explained by these covariates alone. Climate covariates brought additional explanation in the order of 15%, 11%, and 5% on average in the three groups, respectively raising their total average explained variance to 32%, 40%, and 24%. The largest increments in the variance explained were in Texas (29%), South Dakota (22%), and Florida (20%), indicating that climate plays a significant role in modulating the annual tornado frequency in these states. Based on the significant covariates from the model, we can infer that in Texas, all except SOI play a role, in South Dakota, it is primarily from NAO and AMO, and in Florida, NAO, AMO, PDO, and AO feature as the control variables. Similar inferences can be drawn for other states using Table S3, which shows the *p-values* for all the response coefficients.

**Figure 4:**
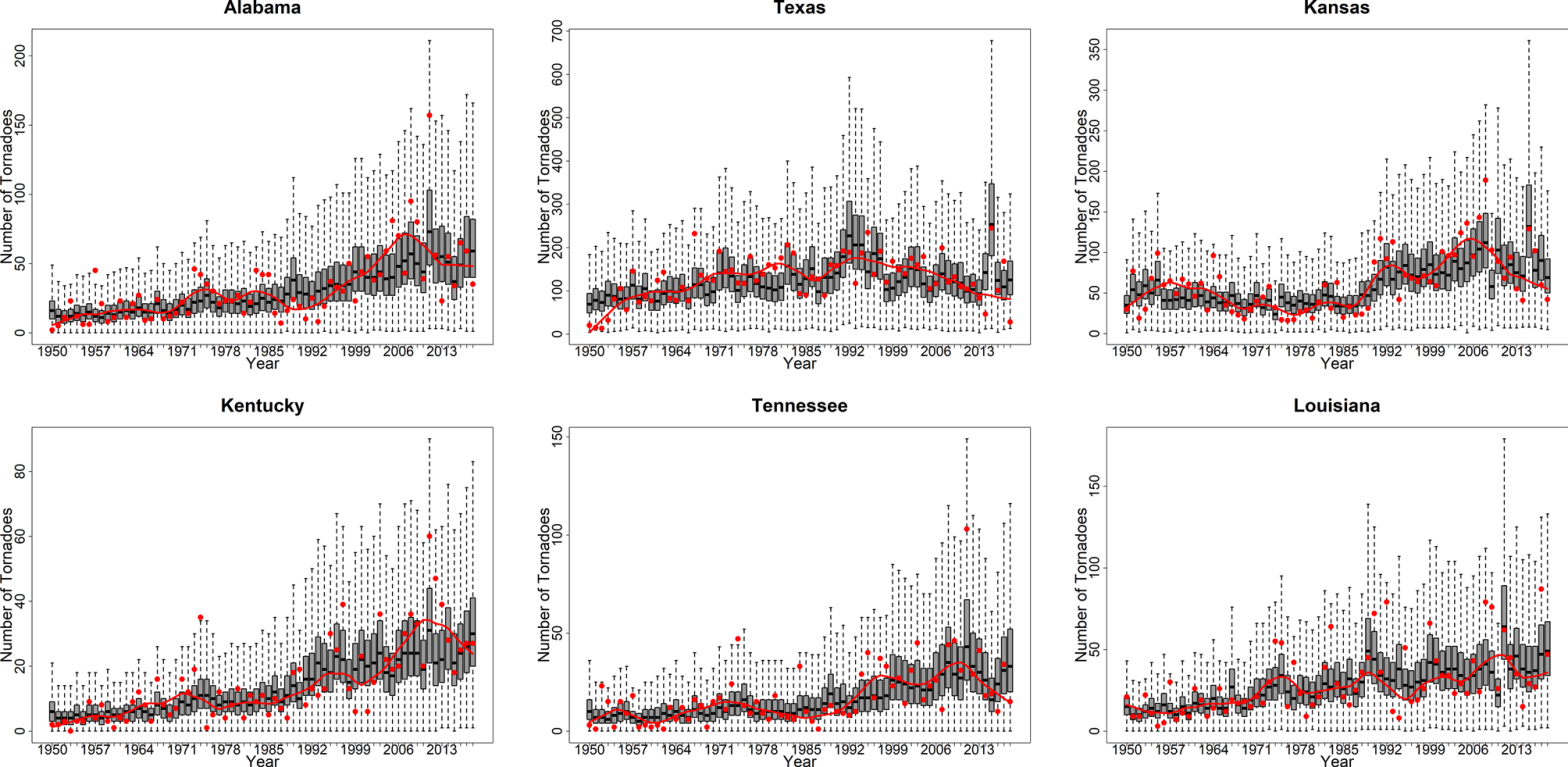
Boxplot of posterior distribution of tornado counts in Alabama, Texas, Kansas, Kentucky, Tennessee and Louisiana. The median of posterior distribution in a state can be referred to the estimated annual tornado counts in that state. The red circles and red line represent annual records of observed tornado counts along with an 11-year low-pass filter to visualize observed trend.

Overall, the anthropogenic and the climate covariates explained more than 40% variability in the annual tornado frequency in eight states, and more than 30% variability in 11 states. For these states, and Texas (explained variance = 33%), Tennessee (explained variance = 47%), and Louisiana (explained variance = 41%), we present the posterior distribution of the annual tornado frequency in Fig. 4. They are presented as a time series composed of boxplots instead of single points because the tornado counts for each year are estimates of the posterior distribution for those years. The boxplots depict those posterior distributions graphically. The record of observed tornado counts data are also shown (red circles) along with an 11-year low-pass filter to visualize the general trend in the data. Secular trend dominated in Alabama (*R*^*2*^ = 43% from M_1_ vs. 54% from M_2_), Kentucky (*R*^*2*^ = 54% from M_1_ vs. 60% from M_2_), and Kansas (*R*^*2*^ = 40% from M_1_ vs. 54% from M_2_). Climate trend dominated in Texas (*R*^*2*^ = 5% from M_1_ vs. 33% from M_2_). In Tennessee (*R*^*2*^ = 28% from M_1_ vs. 47% from M_2_) and Louisiana (*R*^*2*^ = 25% from M_1_ vs. 41% from M_2_), there was evidence of a mixture of these effects. Across all the states, the hierarchical Bayesian model did well in capturing the secular and cyclical trends while producing the uncertainty intervals.

Texas and Kansas were particularly interesting cases due to an existence of low-frequency oscillation and step change. Texas showed a multidecadal variability signal, which was represented well in the model's posterior distribution. Kansas showed a step increase in the annual tornado counts since the 1990s, which was followed by an increase until the early 2000s and a decrease in tornados recently. In this case, too, the posterior distribution captured the three significant changes, thus indicating the model's ability to predict changes based on the anthropogenic and the climate covariates.

To evaluate the posterior distributions of the annual tornado counts, we also verified the coverage rates within the credible intervals and conducted posterior predictive checks. These results are presented in Table S2 of the Supplemental Material. The posterior predictive distribution of the annual tornado counts was assessed by examining the model’s ability to cover the observed counts (coverage rate) within a 95% credible interval^33^. For each state, we computed the coverage rate as the percent number of observations that are inside the 2.5th and 97.5th percentile of the posterior distribution. The average across each of the *Alleys* was approximately 95%, indicating the robustness of the fitted Bayesian models. Following Gelman et al.^34^, we also computed the Bayesian *p*-*value* for two test quantities—the 10th percentile of the data (*y*_*[10]*_) and the 90th percentile of the data (*y*_*[90]*_). Bayesian *p*-*value* is defined as the probability that the replicated data (*y*_*rep*_) could be more extreme than the observed data (*y*) as measured by the test quantities, i.e., $Pr(T({y}_{rep},\theta )\ge T(y,\theta )|y)$. The mean *p-value* for each of the states is presented in Table S2. The tail area probabilities in all the cases were within 0.05 and 0.95, indicating that the model can replicate the observed data well. These verifications confirmed our confidence in interpreting the results and our inference of the response parameters in the model.

**Figure 5:**
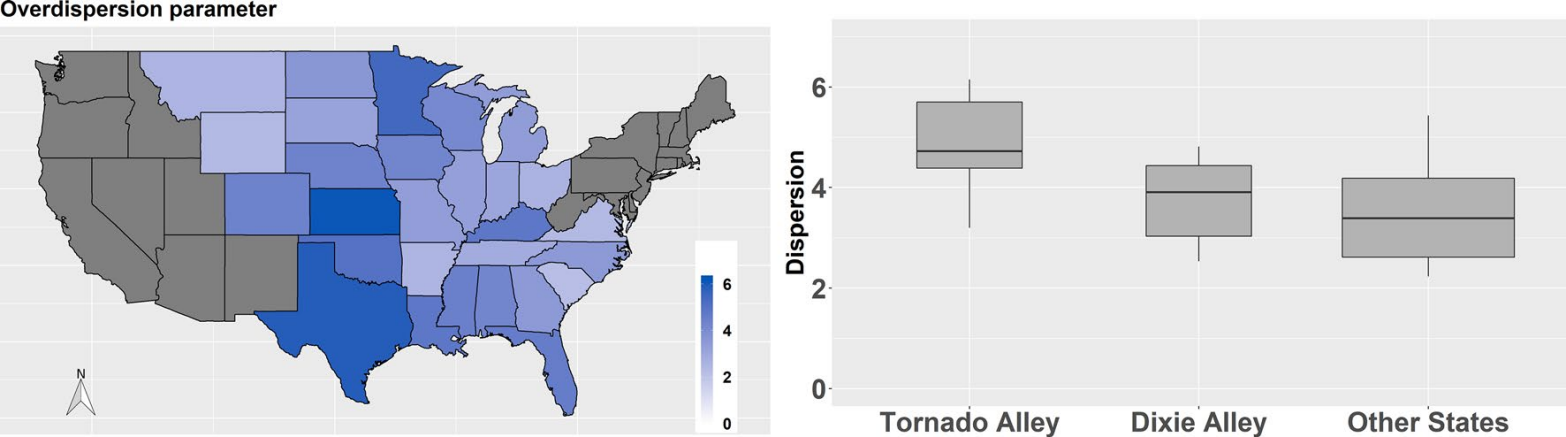
Map of median overdispersion parameter from the negative binomial distribution along with its group-level distribution in *Tornado Alley*, *Dixie Alley* and *Other States*. (The map is created in R version 3.5.2 using the ggplot2 package^32^, https://CRAN.R-project.org/package=ggplot2).

Next, we present overdispersion in the data as modeled using *r*, the overdispersion parameter (see “Methods” section for details). Figure 5(left-panel) shows the spatial distribution of the median of the posterior distribution of *r*. An overdispersion factor that is close to 1 indicates that the data resembles a Poisson distribution with an expected value equal to the variance. Higher values of *r* indicate that the variance is greater than the mean, or that there is more variance (relative to the mean) in the annual tornado counts—a fat tail distribution. All the 28 states had an overdispersion factor that is greater than 2. Kansas, Texas, Minnesota, and Oklahoma were among the states that had a high dispersion factor over 5. We also found that the overdispersion parameter is different in the three groups—*Tornado Alley*, *Dixie Alley*, and *Other States*. Figure 5(right-panel) shows the results of the dispersion parameters for each group as boxplots. The states in *Tornado Alley* had relatively higher dispersion factors compared to those in *Dixie Alley* and *Other States*. The median dispersion factor in the *Tornado Alley* was 4.7, whereas it was 3.9 and 3.4 in the *Dixie Alley* and *Other States*, respectively. *Other States* had more spread compared to the states in *Dixie Alley* with some dispersion factors exceeding 5. It was also interesting to note that among the 13 states with a dispersion factor exceeding 4, the median amount of variance we could explain is at least 40% indicating the model's ability to predict fat-tail events well.

### Inference of the significant predictors

**Figure 6:**
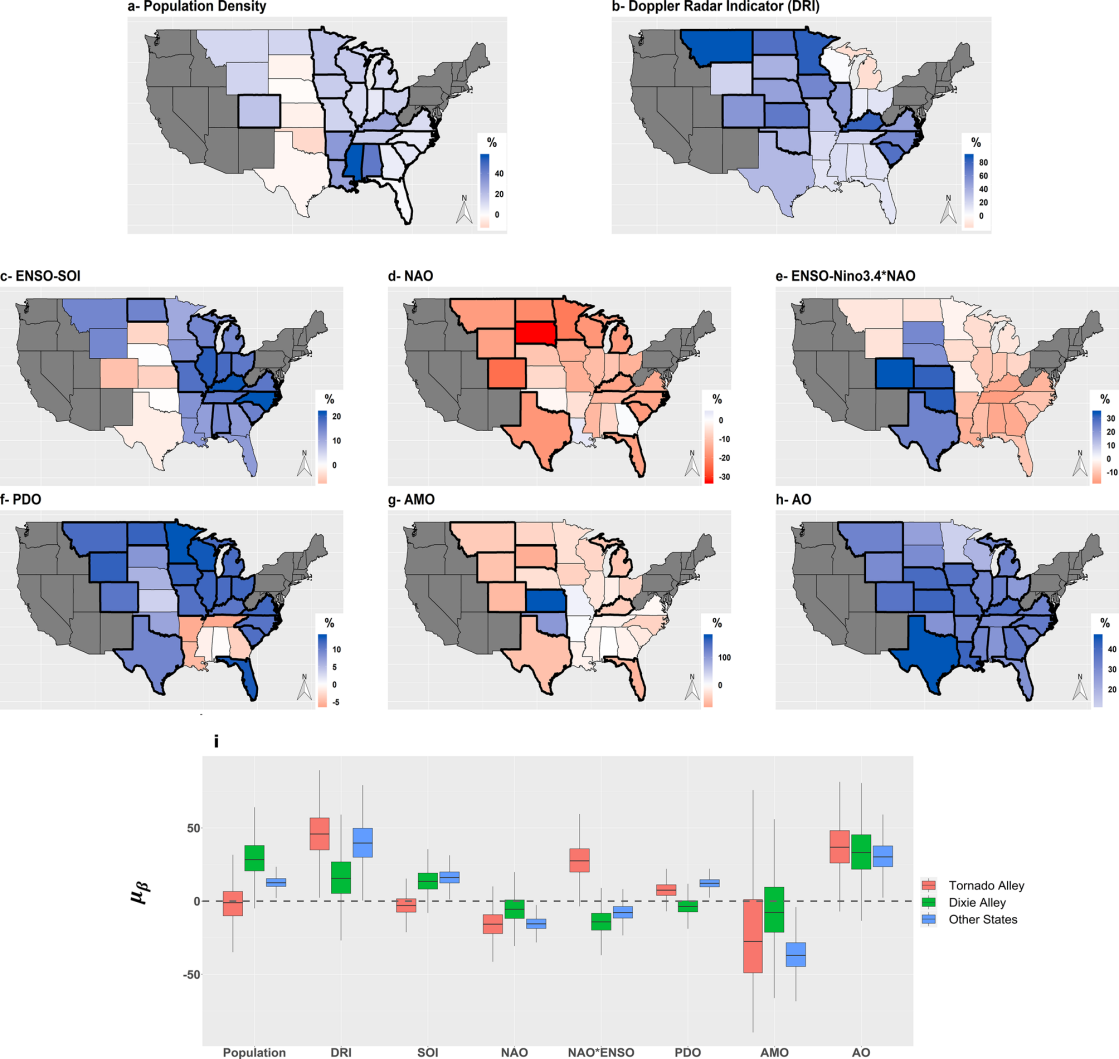
(**a–h**) maps of the regression coefficients expressed as percentage change in the expected annual tornado counts per unit change in the covariate (for figure **a**, population density, we measure change per an increase of 10 people/mi^2^); (**i**) posterior distributions of the mean of the regression coefficients from the hierarchical level as boxplots per the three state-groups. Color scheme in the maps indicates the direction (blue for positive response and orange for negative response); thick boundaries shows the states that have a significant positive or negative association of annual tornado frequency with the corresponding covariate. (Maps are created in R version 3.5.2 using the ggplot2 package^32^, https://CRAN.R-project.org/package=ggplot2).

In Fig. 6, we present the state-level significant covariates (Fig. 6a–h, based on the inference of the regression coefficients ${\beta }_{i\left[k\right]}^{j}$) and their group-level (regional) effects (Fig. 6i, based on the inference of ${\mu }_{{\beta }^{j}}^{k}$). The color scheme in Fig. 6a–h indicates the direction (blue for positive response and orange for negative response) and the strength of association expressed as percentage change in the expected annual tornado counts per unit change in the covariate. A thicker boundary shows states that have *Pr(*${\beta }_{i\left[k\right]}^{j}$ > *0)* > *0.95 or Pr(*${\beta }_{i\left[k\right]}^{j}$ > *0)* < *0.05*. These are the states that have a significant positive or negative association of annual tornado frequency with the corresponding covariate. Figure 6i presents the posterior distributions of ${\mu }_{{\beta }^{j}}^{k}$ (i.e., the mean of the regression coefficients from the hierarchical level) as boxplots per the three state-groups. Full numerical details are presented in Table S3 in the supplemental material.

Population density was a significant predictor in all of the *Dixie Alley* states and much of the *Other States* (Fig. 6a). As expected, it had a positive effect—an increase in the population density is associated with an increase in the rate of annual tornadoes, thus best explaining the secular trend. In the *Dixie Alley* states, there was between 6–57% increase in the rate of annual tornadoes per additional ten people/mi^2^, the largest rate being in Mississippi (57%). In other states, the rate of increase in annual tornadoes ranged from 2% (in Florida) to 27% (in Kentucky) per ten people/mi^2^. Except for Colorado, population density was not a significant predictor in the *Tornado Alley* states. The posterior distribution of the regional coefficient (${\mu }_{\beta }^{population}$, the hierarchical level mean for betas-population) clearly indicates this effect. The regional mean is an indication of the average impact of population density in the respective state-groups. *Dixie Alley* states and *Other States* have a positive mean coefficient at a rate of increase of 28% and 12% per ten people/mi^2^, while the *Tornado Alley* states have a mean in the range of zero.

While the population density was a significant predictor in the *Dixie Alley*, the Doppler Radar Indicator was a significant predictor in the *Tornado Alley* and nine states in the *Other States* category (Fig. 6b). These were the states where step-change due to the Doppler Radars installation in the 1990s best explains the secular trend. In the *Tornado Alley*, there has been a 34–69% increase in the rate of annual tornadoes since the 1990s. Based on the mean of the regression coefficients, we can deduce that regionally, there has been a 46% increase in the rate of annual tornadoes (since the 1990s) in *Tornado Alley*. This increase in the rate is 40% in *Other States*.

The next six panels in Fig. 6 displays the coefficients for the climate covariates. The Southern Oscillation Index (SOI) was a significant predictor in Alabama and Tennessee in *Dixie Alley* and several states from the *Other States* group, but not significant in *Tornado Alley*. The boxplot of the mean of the regression coefficients showing the regional effect further reinforces this inference. However, the interaction term between ENSO and NAO, as treated by Nino3.4*NAO, was significant in four states in *Tornado Alley* (Colorado, Kansas, Oklahoma, and Texas), i.e. ENSO had an impact on the *Tornado Alley* during NAO events. The posterior distribution ${\mu }_{\beta }-ENSO.NAO$ also underlines this finding. On the other hand, NAO had a negative effect, mainly in *Tornado Alley* and *Other States*, not in *Dixie Alley*.

Previous studies on tornado variability also identified ENSO and NAO as significant influence variables. For instance, Cook and Schaefer studied the impact of ENSO phases on the location and strength of U.S. tornado outbreaks from 1950–2003^15^. Based on historical observations, they suggested that sea surface temperature oscillation in the tropical Pacific has different effects on the likelihood of tornado outbreaks depending on the geographic region. El Niño episodes usually limit tornado outbreaks in the Gulf Coast states, including central Florida. At the same time, La Niña affects a larger zone stretching from southeast Texas northward to Illinois, Indiana, and Michigan. Lee and Wittenberg studied springtime ENSO evolution and its potential links to regional tornado outbreaks using records between 1950 and 2014^16^. Their findings showed that in a resurgent La Niña, the probability of a tornado outbreak significantly increased in the Ohio Valley and the upper Midwest. On the other hand, when a two-year La Niña transitions to an El Niño, the risk of a tornado outbreak rises in Kansas and Oklahoma. Marzban and Schafer also examined the correlation between regional tornado activity and sea surface temperature (SST) in four zones in the tropical Pacific Ocean^17^. They found that the strength of correlation in different U.S. regions varied depending on the selected zone in the Pacific Ocean. However, their findings generally identified a strong correlation between cool SST in the central tropical Pacific (La Niña episode) and tornadic activity in an area stretching from Illinois to the Atlantic Coast, and Kentucky to Canada. Allen et al. showed that ENSO influences tornado activity by altering the favorable large-scale environmental conditions such as vertical wind shear and thermodynamic potential energy. Their study revealed that ENSO affects spring and winter tornado occurrences by modulating the position of the jet stream over North America^18^. The study suggested that La Niña causes the atmospheric jet stream to move southeastward, which favors tornado formation in central states due to the temperature gradient. Conversely, El Niño episodes reduce the chances of tornados occurring in the central U.S. due to the weakening of surface winds that typically carry warm and humid air from the Gulf of Mexico. Lee et al.^35^ also discussed that the decay or development of ENSO (Trans-Niño) could led to a pattern of cool temperatures in the central Pacific and warm sea surface temperatures over the eastern tropical Pacific which produced more conducive for spring tornado outbreaks. Cook et al.^36^ also investigated the relationship between sea surface temperature in Nino3.4 region and U.S. tornado outbreaks (in terms of counts and destructive potential) during winter and early spring. Their results showed that La Niña phases are consistently associated with more frequent and stronger tornadoes compared to El Niño conditions. They discussed that this tornado variability is related to the strength and location of subtropical jet during each outbreak and the positions of surface cyclones and low-level jet streams.

In a very recent study, Moore^37^ explored the relationship between seasonal tornado frequency (from 1953 to 2016) and El Niño/Southern Oscillation (ENSO) for multiple U.S. regions. They also examined the spatial dependence of the Tornado Frequency–ENSO relationship and showed that winter and spring tornadoes in the Southeast and Midwest regions were related to ENSO, and the relationship was stronger in winter than in spring in both regions. Mean and median tornado frequencies of the La Nina seasons were notably greater than the other phases' mean and median. They found a significant negative correlation between winter tornado frequency and ONI (anomalies of 3-month running means of sea surface temperature in the Niño 3.4 region) in Southeast and Midwest.

The study by Molina et al.^38^ showed that El Niño, La Niña, and positive phase of SST anomalies in Gulf of Mexico are linked to increased winter tornadoes; El Niño contributed to increased activity in the Southeast U.S., and La Niña increases tornado activity in Midwest and Midsouth. They also reported that tornado favorable conditions were significantly related to anomalously warm and cool SSTs at the Gulf of Mexico. Lepore et al.^39^ investigated the modulation of U.S. convective storms by El Niño–Southern Oscillation and showed that spring tornado activity could be predicted using December-February NINO 3.4 values because ENSO is usually persistent from winter to spring.

The study by Elsner et al.^19^ reported an association between the NAO and tornado activity in the southeast U.S. The findings of this study showed that a positive phase of the NAO (lower than normal pressures over Greenland and higher than normal pressures over the Atlantic) is linked to a lower chance of tornadoes developing across southeastern states, Arkansas, Missouri, and Kentucky. Spencer^40^, a former NASA climate scientist looked into the correlations between the Pacific Decadal Oscillation (PDO) and strong tornadoes, EF3 to EF5. His finding showed that the positive phases of PDO, which was dominant from the mid-1970s until 2005, is associated with fewer tornadoes in the U.S.

The study by Muñoz and Enfield^41^ presented the relation of Intra-Americas low-level jet (IA-LLJ) variability with Atlantic and Pacific climate teleconnections. They calculated Spearman correlations between tornados and the following: NAO, Pacific/North American teleconnection (PNA), Pacific decadal oscillation (PDO), and Niño3.4. The highest correlation achieved between March PNA and March (April) tornado activity is − 0.46 (− 0.33). Their findings revealed that an enhancement of the intra-Americas low-level jet stream during the cold phase of Niño3.4, was linked to an increased occurrence of tornadoes in the east of the Mississippi River. They also reported a connection between negative phase of the PNA pattern during spring and intensification of the IA-LLJ, which could provide greater moisture to the Mississippi and Ohio River basins and lead to increased tornadic activity.

Elsner and Widen^42^ used a Bayesian model to predict seasonal tornado records within a region that stretches across the central Great Plains from northern Texas to central Nebraska. The authors assumed that tornado counts follow a negative binomial distribution and used the sea surface temperatures (SST) over the western Caribbean Sea and Gulf of Alaska during February as predictors. A trend term was also included to account for improvements in tornado reporting over time. The study showed that SST from both regions during February had a significant link to springtime tornado activity across the central Great Plains. There was a 51% and 15% increase in tornado counts per degree Celsius increase in SST of western Caribbean and Gulf of Alaska, respectively. The study showed that the SST covariates explained 11% of the out-of-sample variability in the observed F1–F5 tornado reports. The authors concluded that adding a pre-season covariate for the El Niño, PDO and NAO does not improve their model. Guo and Wang^43^ studied how temporal trends of EF1+ tornadoes varied across the 48 U.S. states during 1950–2013. Their study showed that The Great Plains (including Nebraska and Texas) and Southeast (including Kentucky, Virginia, Tennessee, North Carolina, South Carolina, Alabama, and Georgia) are contributing to the continental‐scale increase in tornado temporal variability.

While our finding regarding ENSO and NAO corroborate previous conclusions based on regional studies, we provided comprehensive state-level summaries through these results using a holistic model. Besides, beyond the findings regarding ENSO and NAO, we also found that PDO events have a significant positive effect in Colorado and Texas in the *Tornado Alley* and all states in the *Other States*, but do not affect *Dixie Alley*. The boxplots of the ${\mu }_{\beta }$ in Fig. 6i is further evidence of this regional effect. AMO, too, modulates the tornado variability in several states in *Tornado Alley* and *Other States*, but not *Dixie Alley*. Similar to NAO, it had a negative effect on the states it influences. Finally, we find that AO modulated the variability in almost all the states and had a positive effect. The positive AO phase enhances the rate of tornadoes in these states. A positive AO phase typically creates a warmer than normal winter in the U.S. through the polar vortex activity^44^ that keeps colder front far north. Warm winters are in turn related to above normal tornado activity. Childs et al.^45^, through a correlation analysis, have previously shown that positive AO phase can be related to enhanced winter season (November to February) tornado activity.

Recognizing that there will be uncertainty in estimating model coefficients due to uncertainties in the input data, especially the choice of where the binary indicator changes from 0 to 1, we also conducted additional experiments to verify the model sensitivity to changes in when this binary indicator shift happens. The model estimation is done for all potential start years from 1991 to 1997. From each of these models, we present in Figure S3, the distributions of the mean of the regression coefficients (${\mu }_{{\beta }^{j}}^{k}$) to verify whether the regional effect changes with change in the input binary variable. We find that the distributions of the mean regression coefficients for the anthropogenic and the climate covariates are similar. While these verification experiments indicate that the model results are robust to changes in inputs, we urge caution in interpreting the coefficients for specific states. We recommend an approach where the model can be updated if new data is available regarding when the Doppler Radar system installation happened in particular states.

## Discussion

Whether the frequency in U.S. tornadoes has changed in the last century had been a tricky question to answer reliably given the changes in the classification of the tornadoes and reporting practices^4,46–49^, and significant and possibly increasing interannual variability inherent in the data^5,50,51^. Verbout et al.^9^ attempted to address this question by fitting linear trend models on individual categories (F/EF scales) in the data for the aggregated counts across the U.S. Others, based on regional analyses, showed that these trends could vary spatially^6,11,13^. However, to our knowledge, a comprehensive assessment of explaining trends and variability across the U.S. by separating secular trends from cyclical variability has not been conducted previously, and hence motivated us to embark on this research of classifying the climatological trends across 28 major tornado-impacted states in the U.S.

In general, this is a high-dimensional problem that offers interesting opportunities since the trends and climate responses have local (state-level) as well as regional (alley-level) effects. A hierarchical Bayesian model with partial pooling was an excellent fit to seamlessly capture these at-state and regional effects. Partial pooling through a hierarchical model also offers a reduction in the uncertainty of the trend/response coefficients. Hierarchical Bayesian models are being used more commonly in applications related to predictions and data description, especially for multivariable problems where the investigator needs to learn something about the group and individual dynamics. Recently, Potvin et al.^52^ used such Bayesian hierarchical modeling framework to estimate tornado reporting rates and expected tornado counts over the central U.S. between 1975–2016 while also addressing spatial non-uniqueness issues. Our study builds on this work and provides an application for seamless estimation of secular and cyclical trends in the annual frequency of tornados across the U.S.

In our models, we attributed the state-level secular trend to two anthropogenic covariates (population density and Doppler radar installation indicator). The regional secular trend was then estimated with the hierarchical model through the pooling of information within the alleys. We attributed the state-level cyclical variability to six climate covariates (SOI, NAO, PDO, AMO, AO, and an interplay term between ENSO and NAO). The common climate response in each of the three alleys was estimated in the partial pooling hierarchical level. By separating the covariates and looking at their difference in the variance explained, we described the effect of large-scale climate in modulating the variability on top of the anthropogenic factors.

We found that, in essence, population density explains the secular trend in *Dixie Alley*. In contrast, the step-change induced due to Doppler Indicator explains the secular trend in *Tornado Alley*. The states in the *Other States* group were affected by a combination of both factors. The secular trend in these states is partly due to population increases and partly due to Doppler radar installation. NAO and the interplay between NAO and ENSO explained the inter-annual to multi-decadal variability in *Tornado Alley*. Further, we found that PDO and AMO are also contributing to this multi-time scale variability. SOI and AO can explain the variability in *Dixie Alley*. In the rest of the *Other States*, interannual to multi-decadal variability was modulated by all the climate covariates, except the interaction term.

These findings regarding PDO, AMO, and AO on the annual frequency of state-level tornadoes across the U.S. are presented here for the first time, and provoke thinking and systematic investigation as to how such low-frequency oscillations may work in modulating the regional variability of tornadoes, and whether the underlying climate processes can suggest, at least qualitatively, that the inference we find here can be explained. Extending this inference model into a seasonal forecast setting using pre-season climate variables^53^ would be of interest too. Such prognostic information is of value to public planners, businesses, and insurance-based risk management agencies^53^. The more we can understand and predict tornado prevalence and occurrence, the more resilience we build to these catastrophic events.

## Methods

### Data

#### Tornados

Historical tornado records were retrieved from the United States (U.S.) Storm Prediction Center (SPC). The SPC's tornado data represents the most reliable accounting of tornado occurrence available over the U.S.^11,42^ This dataset is collected and compiled from National Weather Service (NWS) Storm Data publications and reviewed by the U.S. National Climate Data Center^9,50,54^. The dataset includes information about the date and time, location, path, intensity (Fujita or enhanced Fujita (EF) scale), property losses, crop damages, fatalities, and injuries records on all tornado incidents in the U.S. from 1950 till date. The tornado database can be accessed online at https://www.spc.noaa.gov/wcm/. ^55^ Since the records of tornado occurrence in this dataset is primarily based on eyewitness and reports of storm damages, there might be spatial biases in the data due to the varying population density^56^.

Further, It is widely believed that the number of actual tornado occurrences is greater than the number of reported values, especially before the deployment of the Weather Surveillance Radar-1988 Doppler (WSR-88D)^4,15,57,58^. The primary sources of error are the undetected tornadoes and the lack of consistency in reporting standards. Due to the short-lived and unpredictable nature of tornadoes, they are more likely to be documented if people observe them directly or leave some visual evidence. Accordingly, tornado detection in less populated areas or regions with inadequate communication facilities is challenging. Given that most tornado reports rely on human observations and damage assessments, a region's population has a critical influence on reporting. Normalizing tornado statistics for population bias has been of interest, and several studies have investigated the effect of population change on tornado frequency reports. Snider^59^ looked into the tornado frequency in urban and rural Michigan between 1950–1973 and found a positive correlation between population density and the probability of a tornado being observed and recorded. A study in 1981^60^ estimated that it is extremely hard for a tornado to go unobserved when population density is above 1.5 people per km^2^. Evidence suggests that F2–F5 tornadoes (where F stands for “Fujita scale”) are more likely to be reported due to their greater intensity and longer duration than weaker tornadoes F0–F1. This supports the idea that reports of larger tornadoes are less affected by population density, and the inconsistency/disparity in tornado reports is primarily for F0-F1 categories^57,61^.

The impact of human error on the spatiotemporal variabilities of tornado reports is more emphasized before improvements to the weather radar network. Introduction of the Operational Implement of WSR-88D radars is a key factor contributing to more tornadoes reported since 1990^62^. Since its introduction, the number of tornado-related deaths and personal injuries has decreased by 45 percent and 40 percent, respectively^63^. The emergence of cellular phones, local emergency management offices, and rapid information spread through local media are among other non-meteorological factors contributing to more tornado reports^62^. Less strong tornadoes remain undocumented in sparsely populated regions or areas with insufficient communication infrastructure^49^.

In this study, we considered the annual tornado frequency during 1950–2018 of 28 major tornado-impacted states located in South, Southeast, Ohio Valley, Upper Midwest, and Northern Rockies. Six of these 28 states (Texas, Oklahoma, Kansas, Colorado, Nebraska, and South Dakota) are previously classified as the states that belong to the significant *Tornado Alley*^31,64^. Another six states (Arkansas, Louisiana, Mississippi, Tennessee, Alabama and Georgia) are classified as *Dixie Alley* states^3,30^. A complete list of the 28 states along with their classification into *Tornado Alley* states, *Dixie Alley* states, or *Other States* is presented in Table S1 of the supplemental material. In Figure S1 of the supplemental material, we show the spatial distribution of all the tornadoes during 1950–2018.

#### Population density

Population data for these 28 states were obtained from the U.S. Census Bureau, Population Division. Population counts are collected from the census, which occurs every ten years. Using current data of births, deaths, and migration, the population estimate program (PEP) computes population changes from the latest decennial census, and calculates and updates population count every year. Every annual issuance of population estimates are utilized to revise the entire time series of estimates from July 1, the recent census day of the current year. Full details of the applied methods are found online at https://www2.census.gov/programs-surveys/popest/technical-documentation/methodology/2010-2018/2018-natstcopr-meth.pdf. ^65^ Population density was computed by dividing the population by the state’s area and expressing the values in persons per square miles.

#### Large-scale climate

We used El Niño/Southern Oscillation (ENSO)-Nino3.4, Southern Oscillation Index (SOI), Arctic Oscillation (AO), Atlantic Multi-decadal Oscillation (AMO), Pacific Decadal Oscillation (PDO) and North Atlantic Oscillation (NAO) annual indices to quantify the effect of large-scale climate on the inter-annual variability of tornadoes. Some of these large-scale climate variables were previously found to be significant in explaining the variability in tornadoes^15–19^. We retrieved the monthly time series for these indices from the National Oceanic and Atmospheric Administration's (NOAA's) National Centers for Environmental Prediction (NCEP). Annually averaged indices are used in the model. Several recent studies explored the connection between tornado activity and other climate oscillations such as Global Wind Oscillation (GWO), Madden–Julian oscillation (MJO), and Pacific/North American Pattern (PNA), which vary from seasonal to sub-seasonal timescales^66–75^. However, these climate variables may not be directly related to our study as their impact is mainly seasonal. Hence, we did not use them for inference.

### Analysis and modeling

#### Principal component analysis and wavelet decomposition

To understand the internal structure in the time series of the annual frequency of tornados across these 28 states, we applied a dimension reduction technique followed by a transformation to the frequency domain.

Given that the data exhibited a high correlation, we first applied robust Principal Component Analysis (rPCA) on the 69 by 28 original data matrix to identify the dominant modes. rPCA is an improved version of the traditional PCA that is better at handling outliers. In this approach, the input matrix is decomposed into its low-rank and sparse components by solving a convex optimization program^21^. Singular value decomposition (SVD) is then applied to the low-rank matrix to obtain the uncorrelated principal components (PCs). Hence, rPCA primarily performs PCA on the input matrix's low-rank component after removing the joint outliers. The importance of each PC is quantified based on the fraction of the variance it represents in reference to the original variance in the data.

Next, we performed a wavelet analysis on the first four PCs (dominant modes) to decipher essential cycles inherent to the data. Wavelet transforms permit an orthogonal decomposition of the dominant modes in the time and the frequency domain^22–25^. It uses base functions (from specific families of oscillatory functions that attenuate to zero) differing in time and frequency resolutions. The localized power spectrum then reveals oscillatory behavior in the time series of the dominant modes.

### Hierarchical Bayesian models

Given that there are clear secular trends and internal variability structure in the annual frequency of tornadoes, we attempted to quantify them using nested models. The first model used two anthropogenic covariates—population density, and a Doppler Radar binary variable to capture the secular trend component. The Doppler Radar binary variable was used to model the significant jump or step-change in the tornado reporting since the 1990s^62^. The second model used these two anthropogenic covariates along with inter-annual to multi-decadal climate variability indices (climate covariates) to capture the full spectrum of secular trend and internal variability. The difference in the variance explained between the two models reveals the additional variance that can be explained by large-scale climate covariates on top of the anthropogenic covariates.

In both the models, we assumed a negative binomial model to represent the annual frequency of the tornadoes in each state. The negative binomial model is appropriate for counts data and is a generalization of the Poisson regression model that accounts for overdispersion. The models are structured using a hierarchical Bayesian regression framework that allows the pooling of information across selected states.

The full hierarchical Bayesian model with anthropogenic and climate covariates is presented here. The model with just the anthropogenic covariates to infer the secular trend is a subset of this model.

Data level:

1 $${y}_{it} \sim NegBin\left({p}_{it}, {r}_{i}\right)$$

$${p}_{it}= \frac{{r}_{i}}{{r}_{i}+{\lambda }_{it}}$$

$${\lambda }_{it}={e}^{\left({\alpha }_{i\left[k\right]} + {\beta }_{i\left[k\right]}^{1}*{PD}_{it} + {\beta }_{i\left[k\right]}^{2}*{DRI}_{t} + {\beta }_{i\left[k\right]}^{3}*{SOI}_{t} + {\beta }_{i\left[k\right]}^{4}*{NAO}_{t} + {\beta }_{i\left[k\right]}^{5}*\left[{{Nino34}_{t}*NAO}_{t}\right] + {\beta }_{i\left[k\right]}^{6}*{PDO}_{t} + {\beta }_{i\left[k\right]}^{7}*{AMO}_{t} + {\beta }_{i\left[k\right]}^{8}*{AO}_{t}\right)}$$

Hierarchical level:

$${\alpha }_{i\left[k\right]} \sim N\left({\mu }_{a}^{k}, {\sigma }_{a}^{k}\right)\quad \forall \quad k\in \left(1, 2, 3\right)$$

$${\beta }_{i\left[k\right]}^{j} \sim N\left({\mu }_{{\beta }^{j}}^{k}, {\sigma }_{{\beta }^{j}}^{k}\right)\quad \forall \quad j\in \left(1, 2, \dots , 8\right); k\in \left(1, 2, 3\right)$$

Priors:

$${r}_{i} \sim U\left(0, 100\right)\quad \forall \quad i\in \left(1, 2, \dots , 28\right)$$

$${\mu }_{a}^{k} \sim N\left(0, 100\right)\quad \forall \quad k\in \left(1, 2, 3\right)$$

$${\mu }_{{\beta }^{j}}^{k} \sim N\left(0, 100\right)\quad \forall \quad j\in \left(1, 2, \dots , 8\right); k\in \left(1, 2, 3\right)$$

$${\sigma }_{a}^{k} \sim U\left(0, 100\right)\quad \forall \quad k\in \left(1, 2, 3\right)$$

$${\sigma }_{{\beta }^{j}}^{k} \sim U\left(0, 100\right)\quad \forall \quad j\in \left(1, 2, \dots , 8\right); k\in \left(1, 2, 3\right)$$

Equation 1 shows that the annual frequency of tornados in each state (${y}_{it}$) is modeled as a Negative Binomial distribution with a success parameter (${p}_{it}$) and an overdispersion parameter (${r}_{i}$). The success parameter (${p}_{it}$) relates to the rate of occurrence (${\lambda }_{it}$), which is informed by regression on the anthropogenic and climate covariates. ${\alpha }_{i\left[k\right]}$ are the regression intercepts for state $i$ that belongs to group $k$, and ${\beta }_{i\left[k\right]}^{j}$ are the regression slopes representing the sensitivity of the frequency of tornadoes to the $j$ covariates.

We considered a hierarchical structure for estimating the regression intercept and slope parameters to allow for the pooling of information across states and reducing the associated uncertainty. We classified the states into three groups ($k\in \left(1, 2, 3\right)$)—*Tornado Alley*, *Dixie Alley*, and *Other States*—based on the relative frequency of tornado occurrence. The mean annual number of tornadoes during 1950–2018 in the *Tornado Alley*, *Dixie Alley*, and *Other States* is 58, 27, and 22, respectively. For each of these three groups (*k*), ${\alpha }_{i\left[k\right]}$ and ${\beta }_{i\left[k\right]}^{j}$, were presumed to be drawn from a common distribution whose parameters are, in turn, described by a set of hyperparameters. For instance, ${\beta }_{i\left[1\right]}^{j}$ for the six States in the Tornado Alley ($k=1$) will have a common mean ${\mu }_{{\beta }^{j}}^{1}$ and variance ${\sigma }_{{\beta }^{j}}^{1}$. This representation for each group allows partial pooling across the  states in the group by shrinking the estimates of ${\alpha }_{i\left[k\right]}$ and ${\beta }_{i\left[k\right]}^{j}$ toward a common mean ${\mu }_{{\beta }^{j}}^{k}$ with dispersion parameter ${\sigma }_{{\beta }^{j}}^{k}$, estimated as part of the solution^76^. We assume a non-informative uniform prior on ${r}_{i}$, ${\sigma }_{\alpha }^{k}$, and ${\sigma }_{{\beta }^{j}}^{k}$, and a non-informative normal prior on ${\mu }_{\alpha }^{k}$ and ${\mu }_{{\beta }^{j}}^{k}$.

Using the above model structure, we ran two models, M_1_ and M_2_. M_2_ is the model described above. M_1_ had the same model structure, except that the covariates are only ${PD}_{it}$ and ${DRI}_{t}$ to explain/capture the secular trend. For each model, the parameters were estimated using JAGS version 4.3^77,78^, which employs the Gibbs sampler, a Markov Chain Monte Carlo (MCMC) method for simulating the posterior probability distribution of the parameters. We simulated six chains starting from random initial values for the parameters to verify the convergence of the posterior distribution based on the shrink factor suggested by Gelman and Rubin^79^. The shrink factor compares the variance in the sampled parameters within the chains and across the chains to describe the improvement in the estimates for an increasing number of iterations.

Each chain was run for a 1000 cycle burn-in to discard the initial state, followed by 4000 iterations in the adaptation phase and 25,000 samples of model parameters. The Rhat values for all the parameters are less than 1.1.

We used R version 3.5.3 (https://www.R-project.org/)^80^ and JAGS library version 4.3^77,78^ to run the model. The R and JAGS 4.3 codes with detailed instructions and the relevant data to implement the above-described simulation can be found at http://doi.org/10.5281/zenodo.4317823. Access will be given upon reasonable request.

## Supplementary Information

Supplementary Information
