# Supplementary material for: Explaining the trends and variability in the United States tornado records using climate teleconnections and shifts in observational practices
Source: Sci Rep. 2021 Jan 18;11:1741. doi: 10.1038/s41598-021-81143-5 (PMC7814142; doi:10.1038/s41598-021-81143-5)

**Explaining the trends and variability in the United States tornado records using climate teleconnections and shifts in observational practices**

Niloufar Nouri^1*^, Naresh Devineni^1, 2*^, Valerie Were^2^, and Reza Khanbilvardi^1, 2^

^1^The City University of New York (City College), Department of Civil Engineering, New York, NY 10031, United States

^2^NOAA/Center for Earth System Sciences and Remote Sensing Technologies (CESSRST), The City University of New York (City College), New York, NY 10031, United States

^*^Corresponding Authors: Niloufar Nouri, [nnouri@ccny.cuny.edu](mailto:nnouri@ccny.cuny.edu) ; Naresh Devineni, [ndevineni@ccny.cuny.edu](mailto:ndevineni@ccny.cuny.edu)

**Supplementary Table 1**

Table S1. The list of the major tornado affected states, their classification into *Tornado Alley*, *Dixie Alley*, and *Other States*. The Mann-Kendall Sen’s slope and its *p-value* for the trend test on the annual frequency of tornados is also shown.

|  | **State** | **Alley Name** | **Mann-Kendall Sen's slope (*p-value*)** |  |
| --- | --- | --- | --- | --- |
|  | Colorado | Tornado Alley | 6.4/decade (< 0.01) |  |
|  | Kansas |  | 7.0/decade (< 0.01) |  |
|  | Nebraska |  | 3.9/decade (< 0.01) |  |
|  | Oklahoma |  | *1.6/decade (0.36)* |  |
|  | South Dakota |  | 1.7/decade (0.03) |  |
|  | Texas |  | 9.1/decade (< 0.01) |  |
|  | Alabama | Dixie Alley | 6.7/decade (< 0.01) |  |
|  | Arkansas |  | 3.2/decade (< 0.01) |  |
|  | Georgia |  | 2.5/decade (< 0.01) |  |
|  | Louisiana |  | 4.3/decade (< 0.01) |  |
|  | Mississippi |  | 5.6/decade (< 0.01) |  |
|  | Tennessee |  | 3.1/decade (< 0.01) |  |
|  | Florida | Other States | 5.9/decade (< 0.01) |  |
|  | Illinois |  | 5.4/decade (< 0.01) |  |
|  | Indiana |  | 1.5/decade (0.05) |  |
|  | Iowa |  | 5.6/decade (< 0.01) |  |
|  | Kentucky |  | 3.8/decade (< 0.01) |  |
|  | Michigan |  | *0.5/decade (0.38)* |  |
|  | Minnesota |  | 5.2/decade (< 0.01) |  |
|  | Missouri |  | 3.9/decade (<0.01) |  |
|  | Montana |  | 0.8/decade (< 0.01) |  |
|  | North Carolina |  | 3.3/decade (< 0.01) |  |
|  | North Dakota |  | 3.8/decade (< 0.01) |  |
|  | Ohio |  | 1.7/decade (< 0.01) |  |
|  | South Carolina |  | 2.2/decade (< 0.01) |  |
|  | Virginia |  | 1.6/decade (< 0.01) |  |
|  | Wisconsin |  | 1.6/decade (< 0.01) |  |
|  | Wyoming |  | 0.9/decade (< 0.01) |  |
|  |  |  |  |  |

**Supplementary Figure 1**


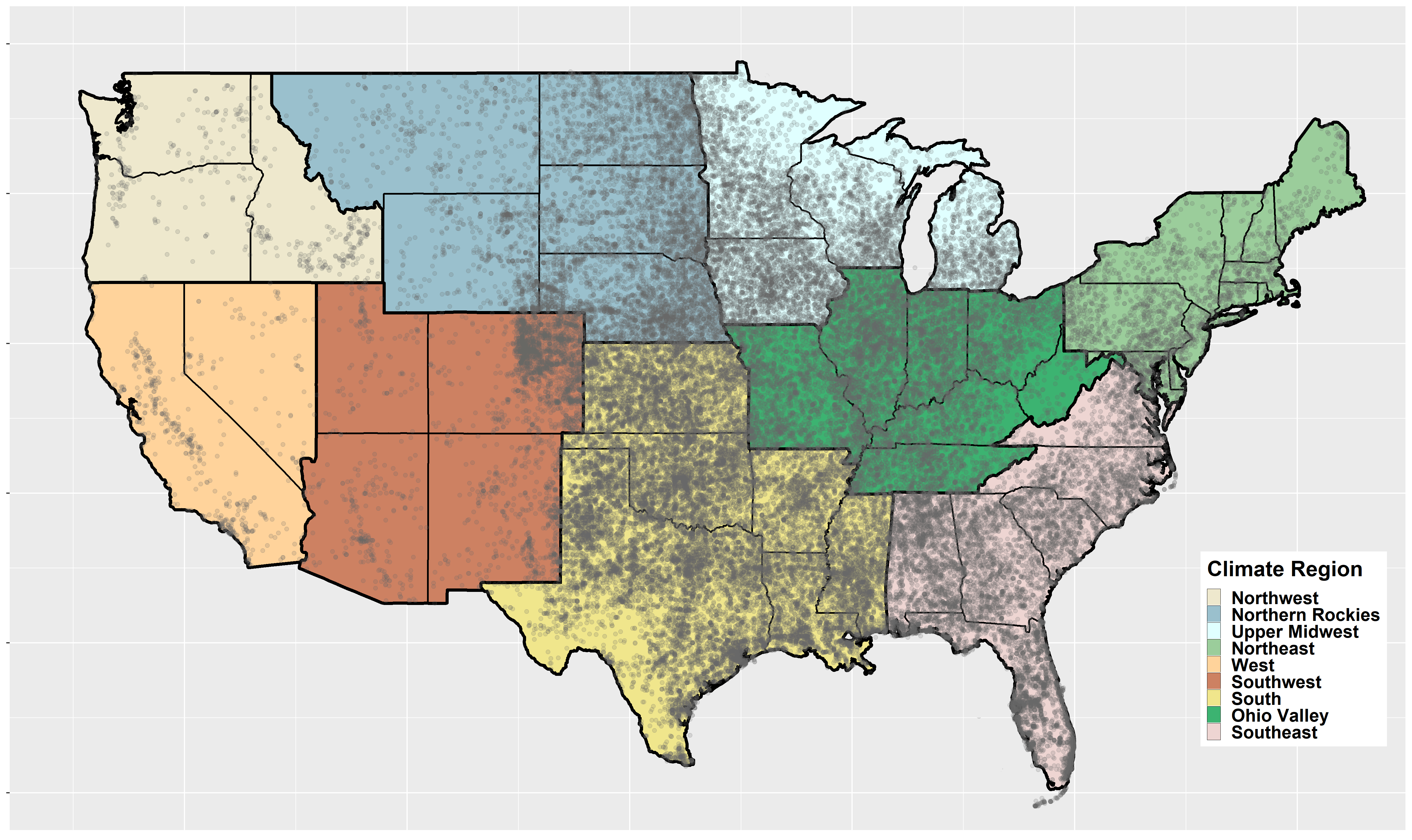


Figure S1. The spatial distribution of all tornado occurrences during 1950-2018. Each colored region represents one of the nine climatically consistent regions identified by National Centers for Environmental Information of NOAA^18^. (The map is created in R version 3.5.2 using the ggplot2 package, https://CRAN.R-project.org/package=ggplot2)

**Supplementary Figure 2**


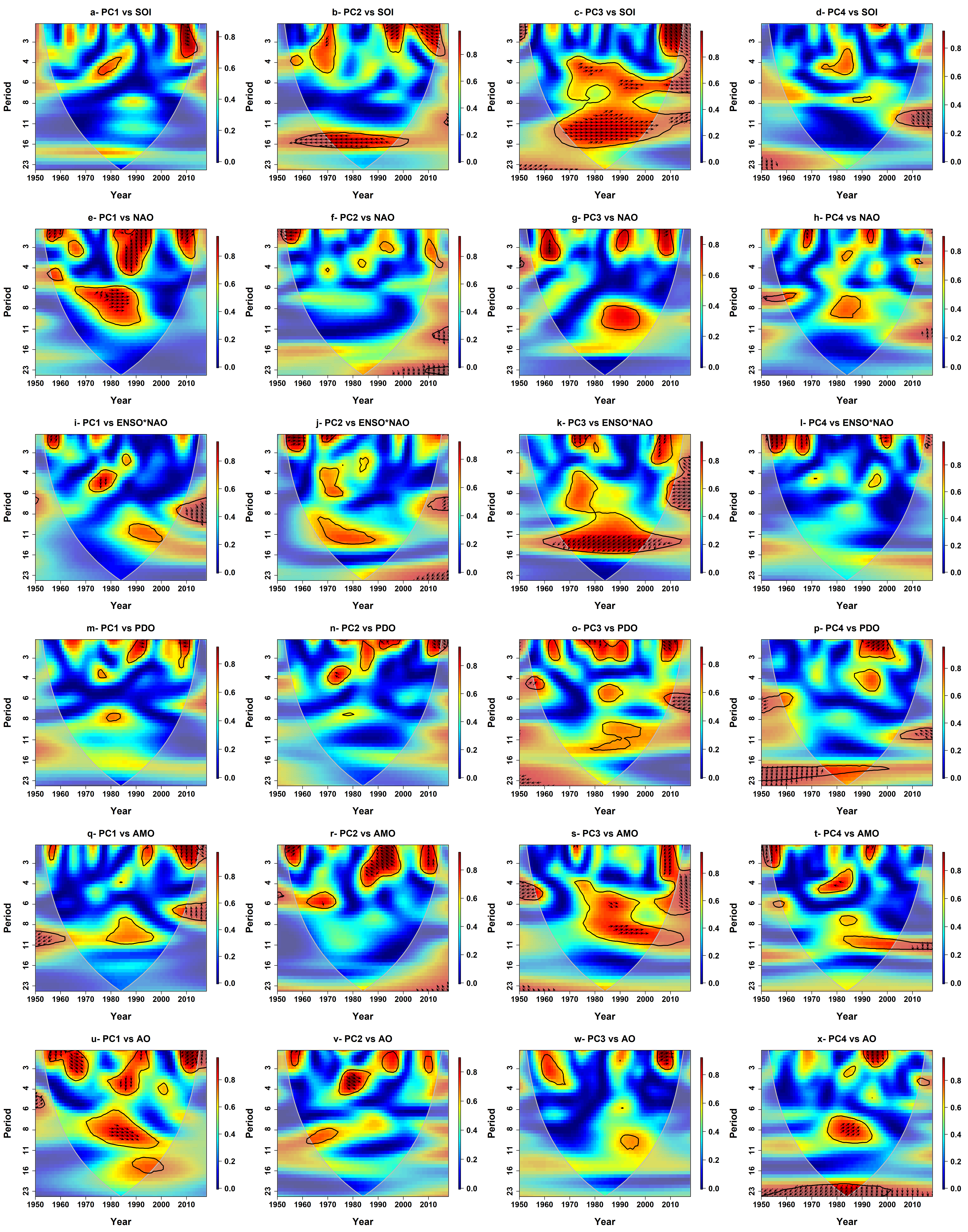


Figure S2. **a-x**, wavelet analysis of coherent structures between scores of the first four principal components and various climate indices, each column shows one PC. **a-d**, wavelet coherence spectrum of first four PCs and SOI, **e-h**, wavelet coherence spectrum of first four PCs and NAO, **i-l**, wavelet coherence spectrum of first four PCs and (ENSO-Nino.34)*NAO, **m-p**, wavelet coherence spectrum of first four PCs and PDO, **q-t**, wavelet coherence spectrum of first four PCs and AMO, **u-x**, wavelet coherence spectrum of first four PCs and AO. Higher wavelet coherence during a specific time shows notable connection between tornado activity and climate index. The areas with solid black line represent significant wavelet coherence at the 90% confidence interval. The arrows show the direction of lag relationship in the data. (Plots are created in R version 3.5.2 using biwavelet package, https://CRAN.R-project.org/package=biwavelet)

**Supplementary Figure 3**


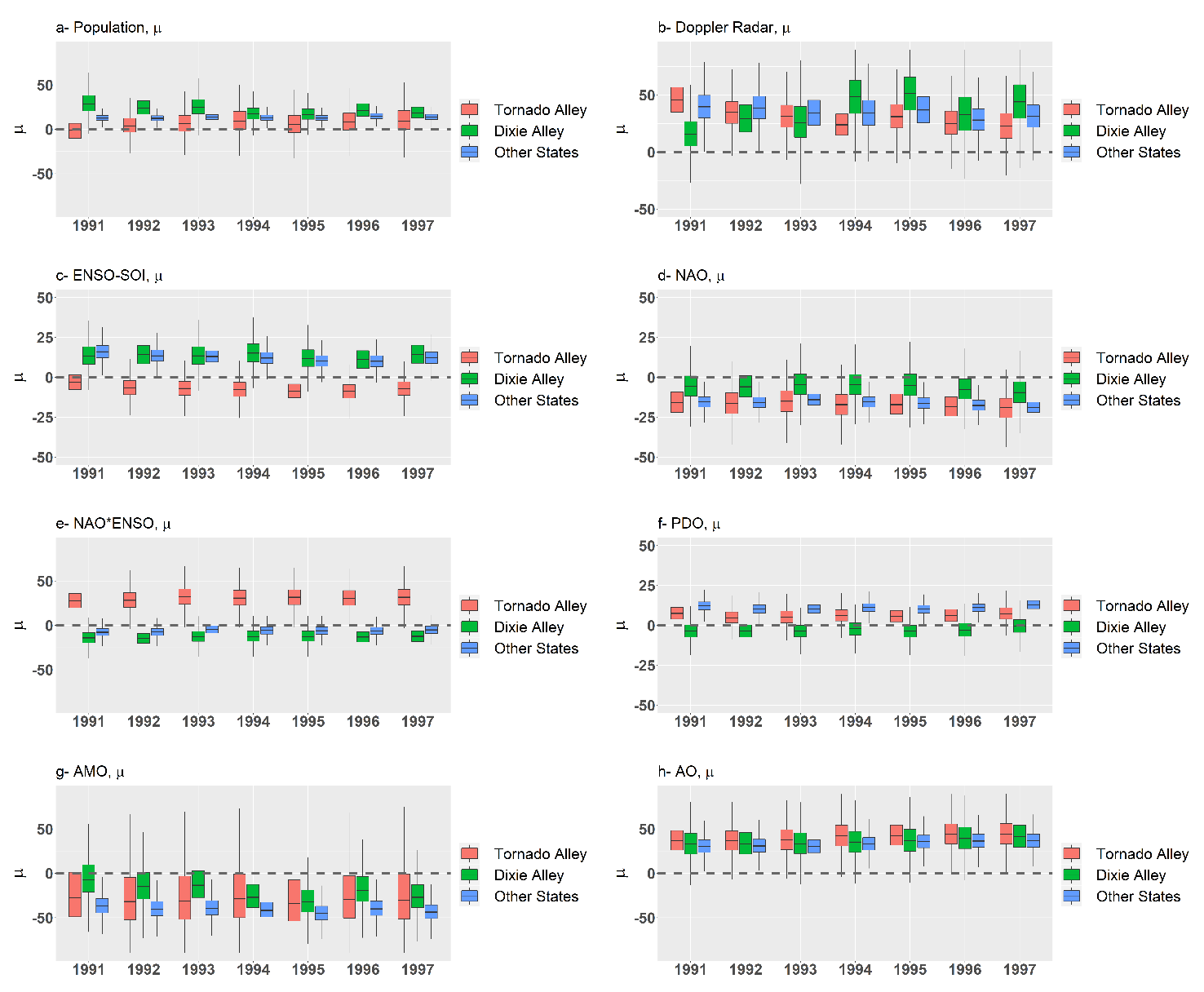


Figure S3. Mean of the regression coefficients from the multiple models estimated using different starting years for Doppler radar installation.

**Supplementary Table 2**


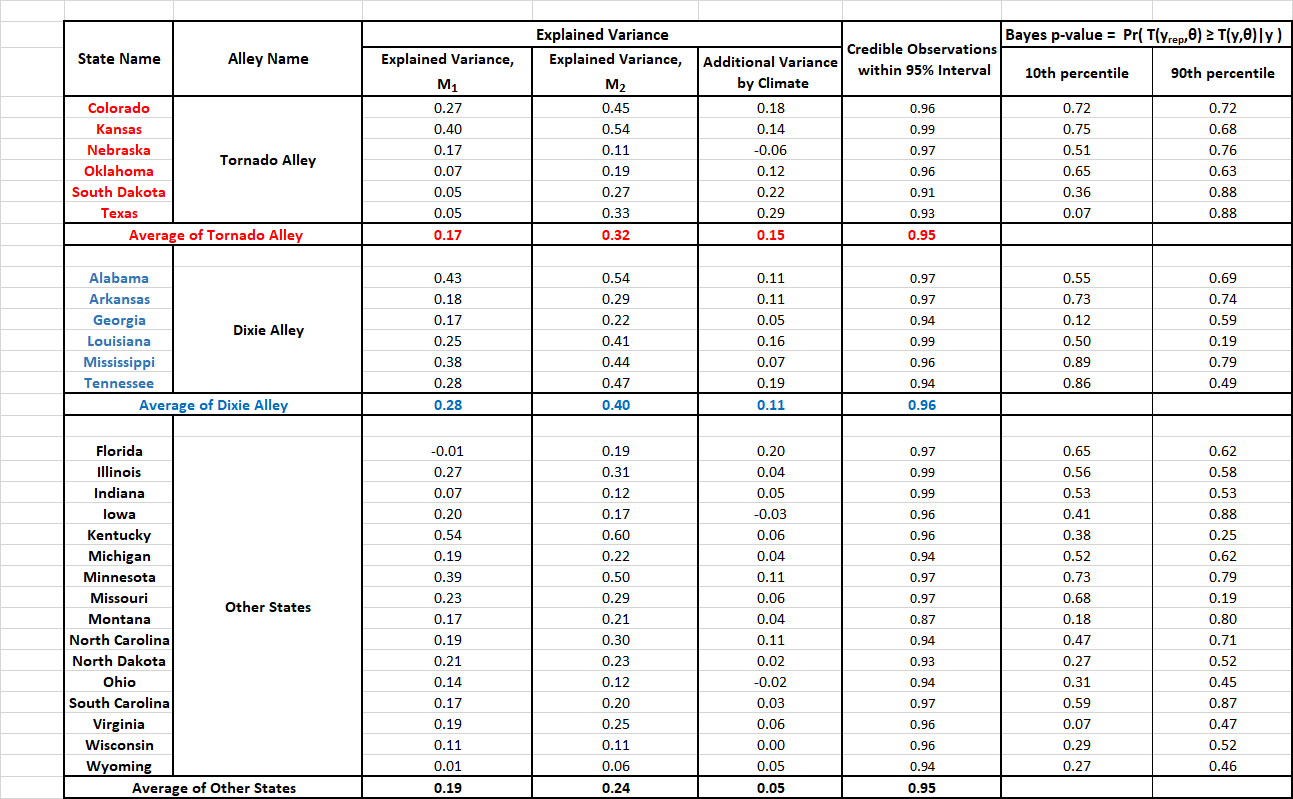
Table S2- Explained variance from each model, coverage rates under the 95% credible intervals, and Bayes *p-value* for M_2_.

**Supplementary Table 3**

Table S3- The posterior median of the regression coefficients for all the 28 states, and the mean of the regression coefficients (hierarchical level) for the three groups of states. Statistically significant coefficients are shown in bold font under the *p-value* column.


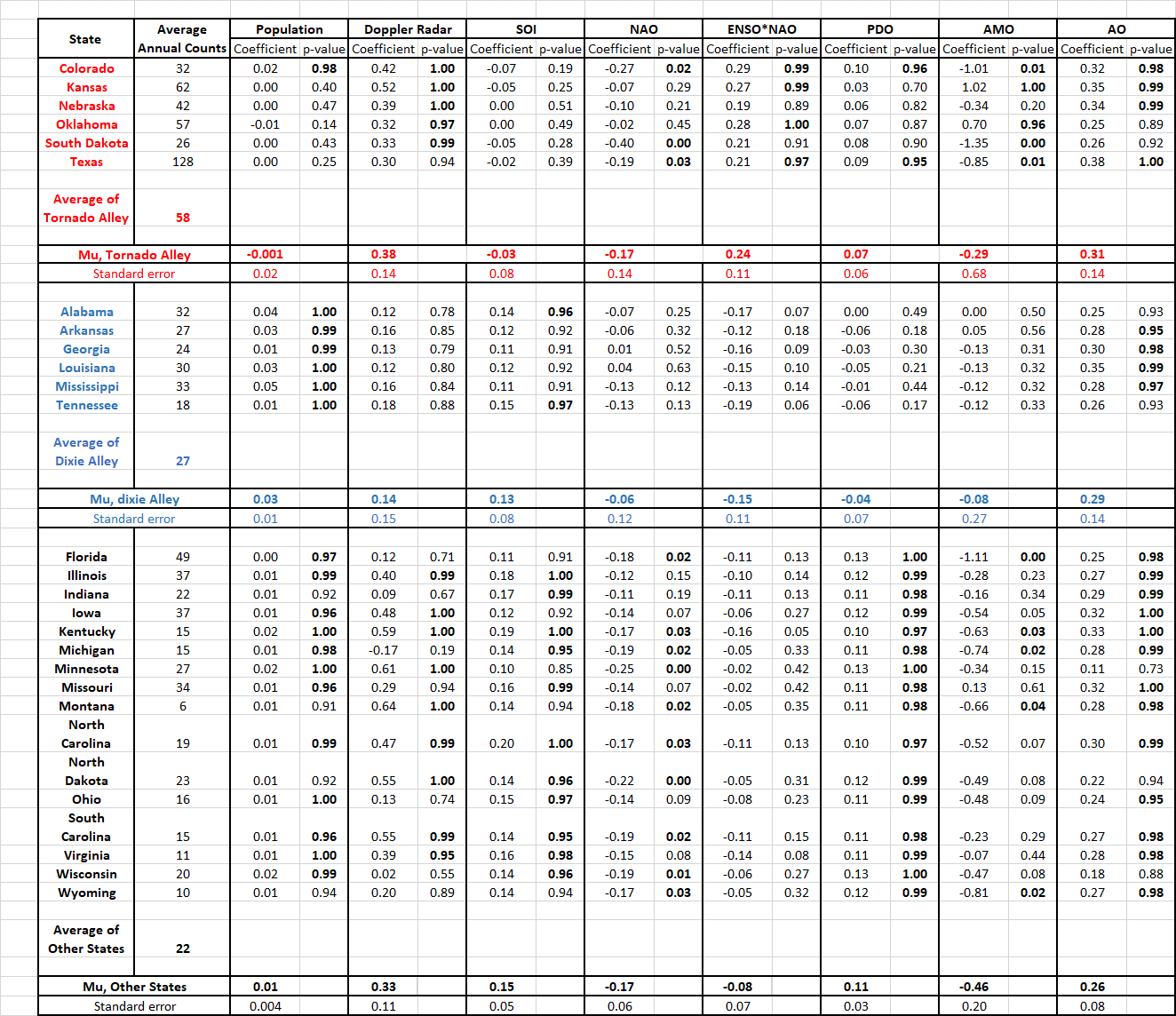

Supplement: Supplementary file 1 — Supplementary Information [file 41598_2021_81143_MOESM1_ESM.docx]
